# Comparison of Predatory Phenotypes and Genotypes Between *Bdellovibrio* sp. BIS2 and *Bacteriovorax* sp. HI3 Isolated From the Same Freshwater Environment

**DOI:** 10.1111/1462-2920.70243

**Published:** 2026-01-22

**Authors:** Tomomi Sugiyama, Tsubasa Kojima, Naoki A. Uemura, Daisuke Nakane, Hideo Dohra, Daisuke Inoue, Michihiko Ike

**Affiliations:** ^1^ Division of Sustainable Energy and Environmental Engineering, Graduate School of Engineering The University of Osaka Osaka Japan; ^2^ Department of Engineering Science The University of Electro‐Communications Tokyo Japan; ^3^ Research Institute of Green Science and Technology Shizuoka University Shizuoka Japan; ^4^ Department of Biological Science, Graduate School of Integrated Science and Technology Shizuoka University Shizuoka Japan; ^5^ Shizuoka Instrumental Analysis Center Shizuoka University Shizuoka Japan

**Keywords:** *Bacteriovorax*, *Bdellovibrio*, *Bdellovibrio* and like organisms, comparative genome analysis, predation phenotype

## Abstract

*Bdellovibrio* and like organisms (BALOs) are predatory bacteria, which play crucial roles in shaping community structure and maintaining diversity in microbial ecosystems. However, functional and genetic variation among coexisting BALOs remains largely unexplored. Here, we characterised *Bdellovibrio* sp. BIS2 and *Bacteriovorax* sp. HI3, which were isolated from the same freshwater environment at different time points. Predation assays and a functional genomic analysis revealed that the predators have distinct prey ranges and predatory behaviours with associated genotypic differences, which may contribute to niche partitioning in the shared habitat. BIS2 possessed an extensive set of flagellar genes, likely contributing to its higher motility and rapid spatial exploration. However, cell debris remained after predation because the prey cells were incompletely lysed. HI3 displayed efficient localised predation and thorough lysis of prey cellular components. The difference in predation efficiency was likely attributed to distinct profiles of hydrolytic enzymes in the two BALOs, including enzymes critical for prey cell lysis and exit from the prey. Genomic regions unique to each predator with predation‐associated genes suggest that genetic divergence drove the evolution of specialised predatory properties across BALOs. These findings provide insights into variation in predatory traits among BALOs, a key mechanism enabling coexistence and adaptation.

## Graphical abstract

*Bdellovibrio* and like organisms (BALOs) have gained attraction owing to their ecological and practical significance. This study comparatively investigated two BALO strains from the same freshwater pond, *Bdellovibrio* sp. BIS2 and *Bacteriovorax* sp. HI3, and revealed significant variation in their predatory behaviours and associated genetic profiles, likely contributing to niche partitioning and enabling BALOs to coexist and adapt in shared habitats.

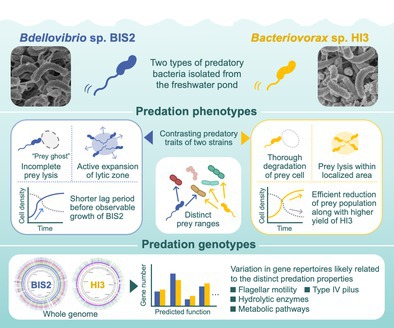

## 1 Introduction

Predation is a fundamental interaction between organisms and a crucial determinant of the structure and dynamics of natural ecosystems. Predator–prey relationships are observed within microbial communities. Predatory bacteria, defined as bacteria capable of killing and consuming other living bacteria as prey (Pérez et al. 2016), are ubiquitously distributed across diverse environments (Linares‐Otoya et al. 2017; Ezzedine et al. 2022). In microbial ecosystems, predatory bacteria play an important role as ecological regulators or balancers; they shape the community structure and maintain microbial diversity through preying on specific bacteria, releasing nutrients into their surroundings and thereby affecting energy flow in the bacterial ecosystem (Hungate et al. 2021; Cohen et al. 2024). Owing to their unique predation abilities and lack of direct harmful effects on higher organisms, practical applications of predatory bacteria in the medical, agricultural, aquacultural and environmental fields have attracted increasing attention (Bratanis et al. 2020; Waso et al. 2021).

*Bdellovibrio* and like organisms (BALOs) are a group of representative obligate predatory bacteria that prey on a broad range of Gram‐negative bacteria and include six genera within the phylum *Bdellovibrionota* (i.e., *Bacteriovorax*, *Bdellovibrio*, *Halobacteriovorax*, *Micavibrio*, *Pseudobdellovibrio*, and *Peredibacter*) (Waite et al. 2020). BALOs are generally found in temperate, neutrophilic, and aerobic environments, and their growth and predation activity are influenced substantially by physicochemical factors, such as temperature, pH, and salinity (Kelley et al. 1997; Inoue, Hiroshima, Nakamura, et al. 2022). BALOs exhibit a unique biphasic life cycle in which a free‐swimming cell infects the prey. Most BALOs, including members of the genera *Bdellovibrio* and *Bacteriovorax*, employ a periplasmic predation strategy. In this mode, a predator attaches to the outer membrane of a prey bacterium and invades the periplasmic space. Then, the predator secretes hydrolytic enzymes to degrade and absorb the host cell contents for its own growth and replication (Pérez et al. 2016). Alternatively, some BALOs adopt an epibiotic predation strategy, wherein the predator attaches to the prey cell envelope and degrades the membrane to digest cellular components externally (Davidov et al. 2006; Deeg et al. 2020; Santin et al. 2024). The prey selectivity and predation efficiency of BALOs are highly diverse, with considerable variation, even among strains within the same genus or species (Jurkevitch et al. 2000; Oyedara et al. 2016; Yu et al. 2017). As BALOs basically depend on prey bacteria as their nutrient source, they have acquired diversified predation properties in response to physicochemical environmental factors and prey species. For instance, the introduction of prey bacteria belonging to different taxa or originating from different environments can affect the structure and diversity of *Bacteriovorax* populations in seawater‐derived cultures significantly (Chen et al. 2012).

Owing to their ecological and practical significance, the functions and underlying mechanisms of predation by BALOs have been studied through various physiological and genetic approaches. In particular, genomic analyses have yielded remarkable insights into the molecular mechanisms underlying BALO predation. Factors determining the variation in predation phenotypes among closely related species and proteins specific to individual predation strategies have been identified for model BALOs, such as *Bdellovibrio bacteriovorus* strains HD100 and 109 J (Pasternak et al. 2014; Banks et al. 2022; Caulton and Lovering 2023). However, despite evidence that BALOs from different genera or species, such as *Bdellovibrio* and *Bacteriovorax*, coexist in the same environments (Wen et al. 2009; Ezzedine et al. 2020), functional and genetic variation among coexisting BALOs remains largely unexplored. To our knowledge, only Oyedara et al. (2016, 2018) have compared predation phenotypes and genetic variation between two different strains of *Bdellovibrio* spp. isolated from the same soil environment. They reported significant intra‐genus differences in predation phenotypes, including differences in plaque formation time and prey range, and characterised critical genomic regions, such as the conserved host interaction locus. However, the precise relationship between phenotypic and genotypic variation remains unclear, underscoring our limited understanding of the heterogeneity of BALO populations in natural environments. Analyses of phenotypic and genotypic diversity among BALOs within a habitat have important ecological and evolutionary implications, providing insight into the mechanisms by which coexisting BALOs partition limited resources to secure individual niches as well as their overall predatory capacity and contribution to the dynamics of the whole microbial ecosystem.

In this study, the predation characteristics of *Bacteriovorax* sp. HI3 (Inoue, Hiroshima, Nakamura, et al. 2022) and *Bdellovibrio* sp. BIS2 isolated from the same freshwater environment were compared. In particular, phenotypic and genotypic characteristics related to predation were analysed extensively through a series of predation assays and pangenome analyses incorporating the two isolates in this study and previously determined BALO genomes. The findings of this study improve our understanding of the genetic basis of the diverse predation phenotypes of BALOs and the mechanisms that enable their coexistence in the aquatic environment.

## 2 Experimental Procedures

### 2.1 Bacterial Strains and Cultivation Conditions

**FIGURE 1:**
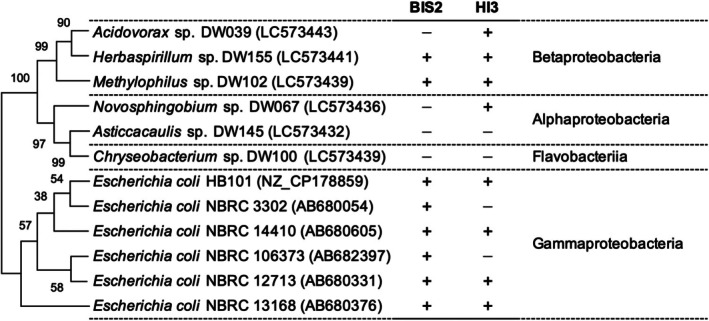
Predation of 12 prey bacterial strains by BIS2 and HI3, as determined using plaque assays. +, predation; −, no predation. A phylogenetic tree based on 16S rRNA gene sequences for prey strains was generated using MEGA X software with the neighbour‐joining method and Kimura 2‐parameter model. Bootstrap values based on 1000 replicates are shown at nodes.

Two BALO strains, *Bacteriovorax* sp. HI3 (Inoue, Hiroshima, Nakamura, et al. 2022) and *Bdellovibrio* sp. BIS2, were used in this study. Both strains were isolated from a freshwater pond at The University of Osaka. Although these strains were obtained at different time points, the coexistence of *Bdellovibrio* and *Bacteriovorax* populations has been repeatedly confirmed in this habitat, suggesting constant coexistence (Inoue, Hiroshima, Ishizawa, and Ike 2022; Saimee et al. 2025). The procedures for the isolation and identification of BIS2 are provided in Texts S1 and S2, and the phylogenetic positions of the two strains within the phylum *Bdellovibrionota* are presented in Figure S1. Twelve Gram‐negative bacterial strains were used as prey bacteria, including six laboratory strains derived from *Escherichia coli* strains K12 and B and six taxonomically different strains (*Acidovorax*, *Novosphingobium*, *Chryseobacterium*, *Methylophilus*, *Asticcacaulis*, and *Herbaspirillum*) isolated from the same freshwater pond as the BALOs (Ishizawa et al. 2020) (Figure 1, Table S1).

HM buffer and HM buffer‐based double‐layer agar plates (Inoue et al. 2023) inoculated with prey bacteria were used for BALO cultivation and predation assays. To prepare the double‐layer agar plate, 100 μL of BALO cell suspension and 300 μL of prey bacterial suspension were mixed with 5 mL of the top layer medium with 0.7% agar. The optical density at a wavelength of 600 nm (OD_600_) of prey bacterial solution was adjusted to 30–50 (final OD_600_: 1.8–3.0 in the soft agar) using a UV‐1850 UV–vis spectrophotometer (Shimadzu, Kyoto, Japan). R2A broth (DAIGO; Shiotani M. S., Hyogo, Japan) was used to culture prey bacteria, excepting *Methylophilus* sp. DW102, for which the medium was supplemented with 2% (v/v) methanol. Agar was added at 1.5% to prepare the solid media. A basal salt medium (BSM) (Inoue, Hiroshima, Nakamura, et al. 2022) was also used as liquid culture for predation assays to accurately assess predation‐induced changes in the dissolved organic carbon (DOC) concentration. Cultivation was conducted at 28°C, and liquid cultures were rotary shaken at 120 rpm.

### 2.2 Preparation of BALO and Prey Cell Suspensions for Predation Assays

To prepare prey cell suspensions, a single colony on an R2A plate was inoculated into 20 mL of R2A broth. After incubation for 1–3 days, cells were harvested by centrifugation (10,000 *× g*, 4°C, 5 min), washed twice, and resuspended in sterile HM buffer or BSM. The concentration (OD_600_) of the suspension was adjusted to the desired level for each experiment.

To prepare BALO suspensions, 2–3 segments (ca. 10 mm^2^ × 4 mm) of the double‐layer agar plate with lytic halos were collected, homogenised using a BioMasher II (Nippi, Tokyo, Japan), and added to the medium with prey *E. coli* HB101. After cultivation for 3 days, the culture was filtered twice through a 0.45 μm filter (Advantec, Tokyo, Japan) to remove residual prey cells and centrifuged (20,000× *g*, 4°C, 10 min) to collect the BALO cells. The pellet was resuspended to give an OD_600_ of 0.001 in 50 mL of HM buffer in which HB101 was inoculated at an OD_600_ of 1.0 in advance. To obtain the active BALO cells, the cultures of BIS2 and HI3 were further incubated for 24 and 40 h, respectively, to reach the exponential growth phase. Then, the culture was again filtered twice through a 0.45 μm filter, and the BALO concentration was adjusted using a quantitative PCR (qPCR) (see Section 2.5) for use in predation assays.

### 2.3 Agar Plate Predation Assay

The top layer of the double‐layer agar plates was prepared by mixing BIS2 or HI3 suspension with prey bacterial suspensions whose OD_600_ was adjusted to 30 for DW102 or 50 for the other prey strains, resulting in a final OD_600_ of 1.8 and 3.0 in the soft agar, respectively. After incubation for 7 days, predation ability was determined by the formation of a lytic halo on the prey lawn. Assays for each BALO‐prey combination were performed in triplicate, and samples without BALO inoculation were used as the negative control.

To further evaluate lytic activity, double‐layer agar plates inoculated with each BALO strain and HB101 as the prey were incubated for 4 days, and the formation, size, and clarity of lytic plaques were monitored over this period. In addition, to observe the predation on prey colonies established on the agar surface, 10 μL of an HB101 suspension in HM buffer (OD_600_ = 1.0) was spotted onto the surface of the R2A agar plate and allowed to air‐dry. Then, 5 μL of a BALO suspension (OD_600_ = 0.001) was spotted 1 cm away from the prey spot. During incubation for 20 days, the lytic behaviour of the BALO on the prey colony was monitored periodically. All assays were performed in triplicate, and samples without BALO inoculation were used as negative controls.

### 2.4 Liquid Culture Predation Assay

To quantitatively evaluate the predation efficiency of BIS2 and HI3, predation assays were conducted in liquid culture using HB101 as the prey. The prey suspension was added to 50 mL of BSM in a 100 mL glass vial to give an OD_600_ of 1.0. Subsequently, the BALO suspension was inoculated to give a BALO concentration of 5.0 × 10^6^ cells/mL, as determined by qPCR. The co‐cultures were then incubated for 7 days. All experiments were performed in triplicate, including the control without inoculating any BALOs. During the incubation period, aliquots (1.5 mL) of the culture were collected at appropriate intervals to monitor predator–prey dynamics. Changes in viable predator and prey cell concentrations were quantified using a viability qPCR. Furthermore, the DOC concentration in the culture was measured using a total organic carbon analyser (TOC‐V_CSH_, Shimadzu) after filtration through a 0.2‐μm filter (Advantec).

### 2.5 Viability qPCR

Viable cells of BALOs and prey bacteria were enumerated using a viability qPCR method adapted from our previous work (Inoue, Hiroshima, Nakamura, et al. 2022). This method employs pretreatment with propidium monoazide (PMAxx) to selectively amplify DNA in viable cells by interfering with PCR amplification of DNA in dead cells. PMAxx (Biotium, Fremont, CA, USA) was added to 200 μL of culture samples at 10 μM. The samples were incubated in the dark at room temperature for 10 min, followed by 15 min of light exposure in an LED Crosslinker 12 (Takara Bio, Shiga, Japan). Thereafter, the cells were pelleted (20,000× *g*, 4°C, 5 min) and washed twice with sterile ultrapure water. DNA was then extracted using a Cica Geneus DNA Extraction Kit (Kanto Chemical, Tokyo, Japan). The qPCR analysis was performed on a CFX Connect Real‐Time PCR Detection System (Bio‐Rad, Hercules, CA, USA) using GeneAce SYBR qPCR Mix α (Nippon Gene, Tokyo, Japan) with 0.2 μM each of forward and reverse primers (Table S2). The thermal cycling conditions were as follows: 50°C for 2 min and 95°C for 10 min, followed by 40 cycles of 30 s denaturation at 95°C, 1 min annealing, and extension at 60°C (HI3), 64°C (BIS2) or 66°C (HB101). Standard curves for quantification were generated using tenfold serial dilutions of standard DNA prepared from PCR products amplified with each primer set (Inoue et al. 2020). The gene copy numbers obtained from qPCR were converted to cell densities based on the copy number of the target gene per genome in each strain (Table S2).

### 2.6 Optical Microscopy

Microscopic observations were performed to assess the appearance of co‐cultures and to measure the swimming speed of BALOs using an upright microscope (BX50; Olympus, Tokyo, Japan) and a CMOS camera (DMK33UX174; The Imaging Source, Bremen, Germany). To observe the appearance of the co‐cultures, samples collected after 96 h of co‐cultivation with HB101 were visualised using an oil‐immersion objective lens (UPlanApo 100 ×, N.A. 1.35, oil immersion; Olympus). A tunnel chamber was assembled by taping coverslips with double‐sided tape (~90 μm thick, NW‐5; Nichiban, Tokyo, Japan). The chamber was pre‐coated with 2% BSA solution and washed with fresh medium. Cell suspension was poured into the chamber, and the ends of the chamber were sealed with nail polish. The cell was visualised using a halogen lamp (U‐LH100L‐3; Olympus) through a bright‐field condenser (U‐AC, NA0.8–0.92; Olympus). To measure swimming speed, the co‐cultures were incubated for 20 h (BIS2) or 30 h (HI3), then filtered through a 0.45 μm filter. The filtrates were visualised using an objective lens (UPlanApo 20 ×, N.A. 0.7; Olympus) with a dark‐field condenser (U‐DCD, NA0.8–0.92; Olympus). Projections of the images were acquired using the imaging software IC Capture (Imaging Source) at 1‐s resolution and converted into an AVI file without compression. All data were analysed by Fiji (ImageJ 1.48v) (Schneider et al. 2012).

### 2.7 Transmission Electron Microscopy (TEM)

A transmission electron microscopy (TEM) analysis was performed to observe the flagellar morphology of BALOs. Samples bound to the grids were stained with 2% (wt/vol) ammonium molybdate and observed by TEM, as previously described (Uemura et al. 2025). The bacterial cell suspension was placed on the carbon‐coated EM grid and incubated for 3 min at room temperature. The cells were chemically fixed with 1% (vol/vol) glutaraldehyde in the motility buffer for 10 min. After washing three times with fresh medium, the cells were stained with 2% ammonium molybdate and air‐dried. Samples were observed under a transmission electron microscope (HT7800; HITACHI, Tokyo, Japan) at 80 kV. The EM images were captured by a camera and analysed by ImageJ 1.54 g.

### 2.8 Comparative Genome Analysis

A comparative genome analysis was performed using the whole genome sequences of HI3 (Inoue, Hiroshima, Ishizawa, et al. 2022) and BIS2, along with those of 22 other strains belonging to *Bdellovibrionota* (genera *Bdellovibrio, Pseudobdellovibrio*, *Bacteriovorax*, *Halobacteriovorax*, *Peredibacter*, and *Micavibrio*) whose genomes were publicly available and listed as complete as of July 2024 (Table S3). Molecular phylogenetic relationships were inferred using a multi‐locus sequence analysis (MLSA). A set of 81 universal bacterial core genes, including 16S rRNA and *rpoB* (which is effective for subdividing phylogenetic groups within BALOs (Piñeiro et al. 2008)), was extracted from each genome using the Up‐to‐date Bacterial Core Genes 2 (UBCG2) pipeline (Kim et al. 2021). Based on the concatenated alignment of these core genes, a maximum likelihood phylogenetic tree was constructed using the GTR + CAT model as implemented in RAxML ver. 8.2.13. The resulting tree was visualised using MEGA ver. X (Kumar et al. 2018). The average nucleotide identity (ANI) was computed for all combinations of 24 strains after aligning the genome sequences using the BLAST algorithm in the JspeciesWS web server (https://jspecies.ribohost.com/jspeciesws/#analyse, accessed in December 2024) (Richter et al. 2016). Pan‐ and core‐genome analyses were conducted across the 24 selected genomes using GET_HOMOLOGUES software ver. 07112023 (Contreras‐Moreira and Vinuesa 2013). Orthologous gene clusters were identified using both OrthoMCL (OMCL) ver. 1.4 (Li et al. 2003) and bidirectional best‐hit (BDBH) algorithms, with a BLASTP *e*‐value threshold of < 0.001 and alignment coverage of 30%–70%. Based on the OMCL clustering results, the softcore‐genome was defined by genes present in 95%–100% of the genomes, shell genes were those present in three or more genomes but less than 95%, and cloud genes were those present in one or two genomes. To identify unique or divergent genomic regions in BIS2 and HI3 relative to their close relatives, pairwise whole‐genome comparisons were performed and visualised using BLAST Ring Image Generator (BRIG) (Alikhan et al. 2011) and Genome Matcher (Ohtsubo et al. 2008) based on nucleotide sequence alignments generated using BLASTN. Potential functions were assessed through annotating predicted coding sequence (CDS) based on Clusters of Orthologous Groups (COG) and KEGG Orthology (KO). Nucleotide sequences of predicted CDS were converted into amino acid sequences and analysed using RPS‐BLAST ver. 2.2.15 (Altschul et al. 1990) and KEGG Automatic Annotation Server (KAAS) ver. 2.1 (Moriya et al. 2007) to obtain COG and KO assignments, respectively. Based on the KO annotations, genes assigned to the flagellar system, pilus system, and hydrolase, which are potentially associated with predation characteristics, were compared between BIS2 and HI3 with respect to presence, absence, and copy number. Additionally, metabolic pathways involved in carbohydrate metabolism and ATP synthesis were reconstructed and compared by utilising the KEGG pathway database (Kanehisa et al. 2004).

## 3 Results and Discussion

### 3.1 Prey Range of *Bdellovibrio* sp. BIS2 and *Bacteriovorax* sp. HI3

The predation abilities of BIS2 and HI3 against 12 candidate prey strains were evaluated using the double‐layer agar plating technique. Both BALOs formed visible plaques on four *E. coli* strains, *Herbaspirillum* sp. DW155 and *Methylophilus* sp. DW102 and did not prey on two environmental isolates (Figure 1). Two *E. coli* strains and two environmental isolates (*Acidovorax* sp. DW039 and *Novosphingobium* sp. DW067) were susceptible to predation only by BIS2 and HI3, respectively, indicating that the two BALOs show distinct predation spectra.

Previous studies have suggested that the prey taxon influences susceptibility to predation by BALOs (Inoue, Hiroshima, Nakamura, et al. 2022). For example, the NpdA‐based S‐layer found in β‐ and γ‐proteobacteria confers protection against *B. bacteriovorus* predation (Koval and Bayer 1997; Aharon et al. 2021; Gajbhiye et al. 2023). A BLASTP search revealed that the genome of *Acidovorax* sp. DW039 (accession no. NZ_AP029019), which resisted predation by BIS2, contains a gene encoding a protein with 54% amino acid identity (99% coverage) to NpdA of *Paracidovorax citrulli* M6. These findings indicate that S‐layer production might enable strain DW039 to escape predation by BIS2. There is well‐documented variation in susceptibility to BALO predation, even among strains belonging to the same genus or species (Dashiff et al. 2011; Oyedara et al. 2016; Kongrueng et al. 2017; Saralegui et al. 2022). In this study, among five *E. coli* K12‐derived strains, HI3 failed to form lytic plaques on strains NBRC 3302 and NBRC 106373. These results imply that even minor genomic differences among prey can influence susceptibility to BALO predation, consistent with earlier results demonstrating that *Vibrio cholerae* and *P. citrulli* lost resistance to *B. bacteriovorus* predation by mutations in a single gene associated with motility, cell membrane components, or secretion systems (Duncan et al. 2018; Aharon et al. 2021).

### 3.2 Lytic Activity on Agar Plates

**FIGURE 2:**
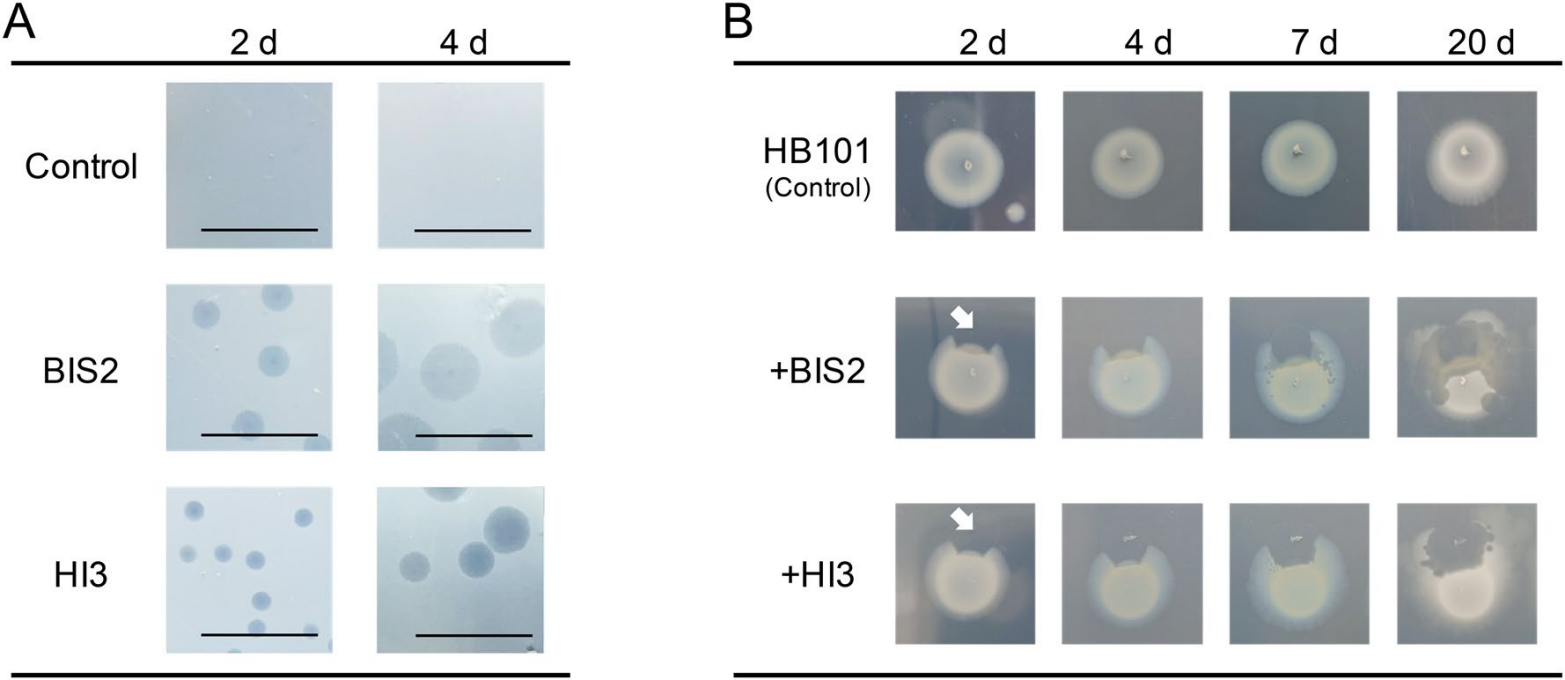
Predation phenotypes of BIS2 and HI3. (A) Plaque growing on a double‐layer agar plate. The scale bar indicates 1 cm. (B) Predation on a bacterial colony on the surface of nutrient agar. White arrow shows the BALO inoculation point.

To compare the lytic activities of BIS2 and HI3, predation assays were conducted in double‐layer agar plates or on the surface of nutrient agar plates, applying *E. coli* HB101 as a prey species. In double‐layer agar plate assays, plaques formed by both strains on prey lawns became visible after 2 days (Figures 2A and S2). By day 4, plaques formed by BIS2 reached an average diameter of 4.3 mm, whereas those formed by HI3 were smaller, averaging 2.7 mm. Another notable difference was observed in plaque clarity; HI3 produced completely clear plaques, whereas BIS2 formed plaques with incomplete clearance. When BIS2 and HI3 suspensions were spotted onto nutrient agar plates pre‐inoculated with HB101 cells, lysis of established prey colonies by both BALOs was observed after 2 days (Figure 2B). However, the lytic zones appeared turbid on high‐density prey colonies, particularly around the original prey spot, indicating residual prey cells or debris. At 7 days after the inoculation of BIS2, smaller lytic spots appeared in distant parts of the inoculation point, even on the opposite side of the main prey colony. In contrast, when HI3 was applied, lytic spots were typically detected near the site of inoculation and became completely transparent. The phenotypic differences in the clearance and expansion of lytic areas became pronounced after 20 days. These contrasting lytic phenotypes were also observed against another prey strain, DW155, confirming that these behaviours are inherent to predators (Figure S3). To investigate the mechanism behind the rapid expansion of BIS2, we directly measured the swimming speeds of BIS2 and HI3 using optical microscopy. BIS2 exhibited significantly higher swimming speed (mean: 90.9 μm/s) than HI3 (mean: 80.2 μm/s) (Figure S4 and Video S1). Although other factors such as swimming patterns or burst size may also influence the expansion of the lytic zone, the higher motility of BIS2 appears to be a key contributor. Consequently, BIS2 likely spread more actively across the prey population but exhibited incomplete prey lysis, whereas HI3 achieved more thorough degradation of prey cells within a smaller, localised area.

### 3.3 Predation Dynamics in Liquid Culture

**FIGURE 3:**
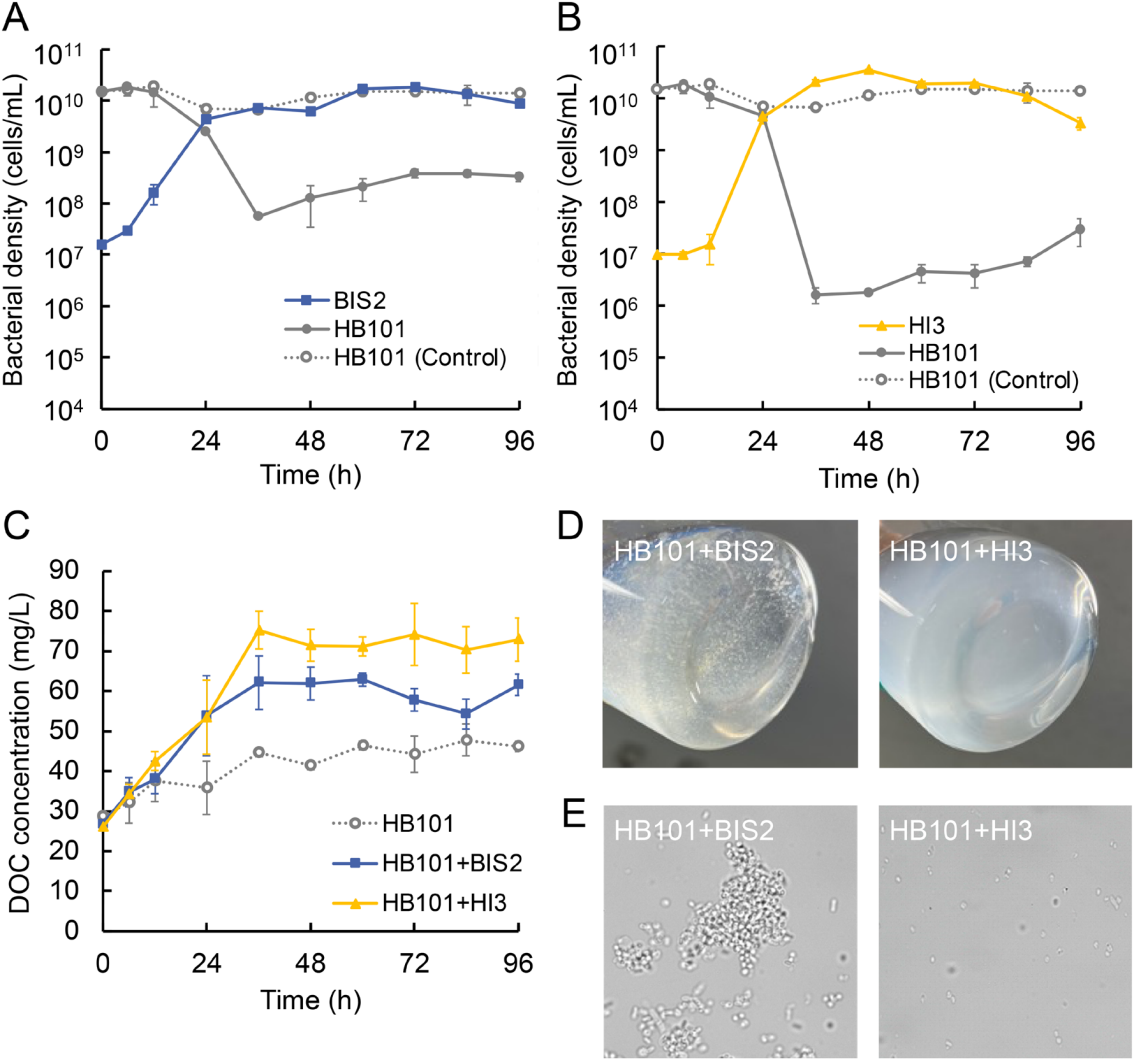
Temporal variation in bacterial cell densities based on viability qPCR during the cultivation of HB101 as prey with (A) BIS2 or (B) HI3. (C) Temporal changes in the DOC concentration. (D) The appearance and (E) optical microscope images (135 μm × 135 μm) of co‐cultivation cultures after 96 h. Error bars in panels (A–C) represent standard deviations (*n* = 3).

To investigate BIS2 and HI3 predation dynamics, co‐culture experiments were conducted in a liquid medium using HB101 as prey. Without inoculating any BALO strains, the HB101 cell density remained stable at 0.7–1.6 × 10^10^ cells/mL throughout the experimental period (Figure 3A,B). When BIS2 and HB101 were co‐cultivated, the prey density remained stable at ca. 1.5 × 10^10^ cells/mL for the first 12 h and then declined rapidly to 2.5 × 10^9^ cells/mL by 24 h and to 5.5 × 10^7^ cells/mL (99.63% decline) by 36 h (Figure 3A). BIS2 (with an initial density of 1.5 × 10^7^ cells/mL) showed population doubling by 6 h. The cell density of BIS2 reached 4.2 × 10^9^ cells/mL at 24 h and peaked at 1.7 × 10^10^ cells/mL at 72 h. In the co‐culture of HI3 and HB101, the prey population began to decrease by 6 h and was reduced to 1.6 × 10^6^ cells/mL (99.99% decline) by 36 h (Figure 3B). HI3 initially exhibited slower growth than that of HB101, increasing ca. 1.5‐fold in the first 12 h, after which growth accelerated dramatically, reaching a peak density of 3.5 × 10^10^ cells/mL at 48 h. These results demonstrated the prey‐dependent growth of both BALOs and confirmed differences in predation behaviours. In particular, BIS2 had a shorter lag period before initiating vigorous predation, whereas HI3 ultimately reduced the prey population to a much lower level and achieved a higher peak density. Both BALO populations declined gradually following their peaks, particularly for HI3, showing a decrease of over tenfold. The reduction of BALO populations likely resulted from the depletion of prey cells, leading to inefficient predation. The DOC concentration in the co‐cultures increased by ca. 20 mg/L within 24 h compared with that in the control without inoculating BALOs (Figure 3C), suggesting the release of lysed cell components generated via predation. In contrast, the HB101 population recovered to nearly 10^7^–10^8^ cells/mL after 36 h, together with a gradual decrease in the DOC concentration, indicating that regrowth occurs through utilising solubilised cellular components. This phenomenon has also been observed in previous studies (Shemesh and Jurkevitch 2004; Williams et al. 2019; Inoue, Hiroshima, Nakamura, et al. 2022).

Notably, in the co‐culture inoculated with BIS2, white suspended matter was observed during active predation and remained visible even more than 10 days after the co‐culture experiments (Figure 3D). This cell debris was clearly observed under optical microscopy (Figure 3E). Additionally, the increase in the DOC concentration was considerably lower after 36 h in the co‐culture with BIS2 (ca. 40% lower) than in the co‐culture with HI3. In contrast to this significant difference in DOC, the total number of prey cells consumed was comparable between the two predators (BIS2: 1.5 × 10^10^ cells/mL, HI3: 1.6 × 10^10^ cells/mL). Theoretically, if both predators solubilised the prey cellular components with equal efficiency, the released DOC levels should be similar. Therefore, the lower DOC increase in the BIS2 co‐culture strongly suggests that BIS2 leaves a substantial portion of prey biomass as insoluble debris, whereas HI3 achieves more thorough solubilisation. These findings suggested that the suspended matter in the co‐culture of BIS2 and HB101 is likely the remnant of incompletely lysed prey cells (i.e., prey ghost) generated through predation by BIS2. The incomplete prey lysis in liquid culture was consistent with turbid plaques and residual prey found in agar plate assays (Figures 2 and S3). Thus, BIS2 might exit the bdelloplast before the complete degradation of prey cells. This strategy could potentially allow it to rapidly enter a free‐swimming (host seeking) phase, although other factors, such as the specific resistance of prey cell wall structures to BIS2 hydrolytic enzymes, cannot be entirely ruled out. This predation strategy could explain the short lag period prior to observable growth (Figure 3A) and active expansion of the lytic zone (Figure 2B). A recent study applying a mathematical model simulation proposed a rate versus yield trade‐off in bacterial predation, allowing the coexistence of a “fast” and “high affinity” predator (Summers and Kreft 2022). Our results support the potential for such a coexistence strategy of multiple BALOs in the same environment. Aggregates of incompletely lysed prey cells could act as decoys for BALO predation. BALOs recognise prey envelopes via type IV pili (TFP) and typically avoid invading dead cells (Rotem et al. 2015). An increase in decoys might reduce the contact of BALOs with viable prey. This could explain the smaller reduction in the prey population by BIS2 than by HI3, particularly from 36 h onwards. While the current study established their individual baselines, future co‐cultivation experiments are needed to clarify whether these contrasting strategies allow for their stable coexistence in a shared environment.

### 3.4 Comparative Genomics of BIS2, HI3 and Other BALOs

Analyses of 24 BALO strains including BIS2 and HI3 revealed generally low genomic similarity with pairwise ANI values of < 93%, except between BIS2 and SSB218315 (97.4%) and between the other three closely related pairs (Figure S5). A pangenome analysis of these genomes identified 16,384 gene clusters using the OMCL algorithm (Figure S6A) and 11,882 gene clusters using the BDBH algorithm (Figure S6B). Core clusters shared by all 24 genomes accounted for only 2.5% of the total gene clusters found using each algorithm. Furthermore, nearly half of the total gene clusters found using the OMCL algorithm were identified as strain‐specific clusters (Figure S6A, Tables S4 and S5), indicating significant genetic variation among BALOs (Davis et al. 2024).

**FIGURE 4:**
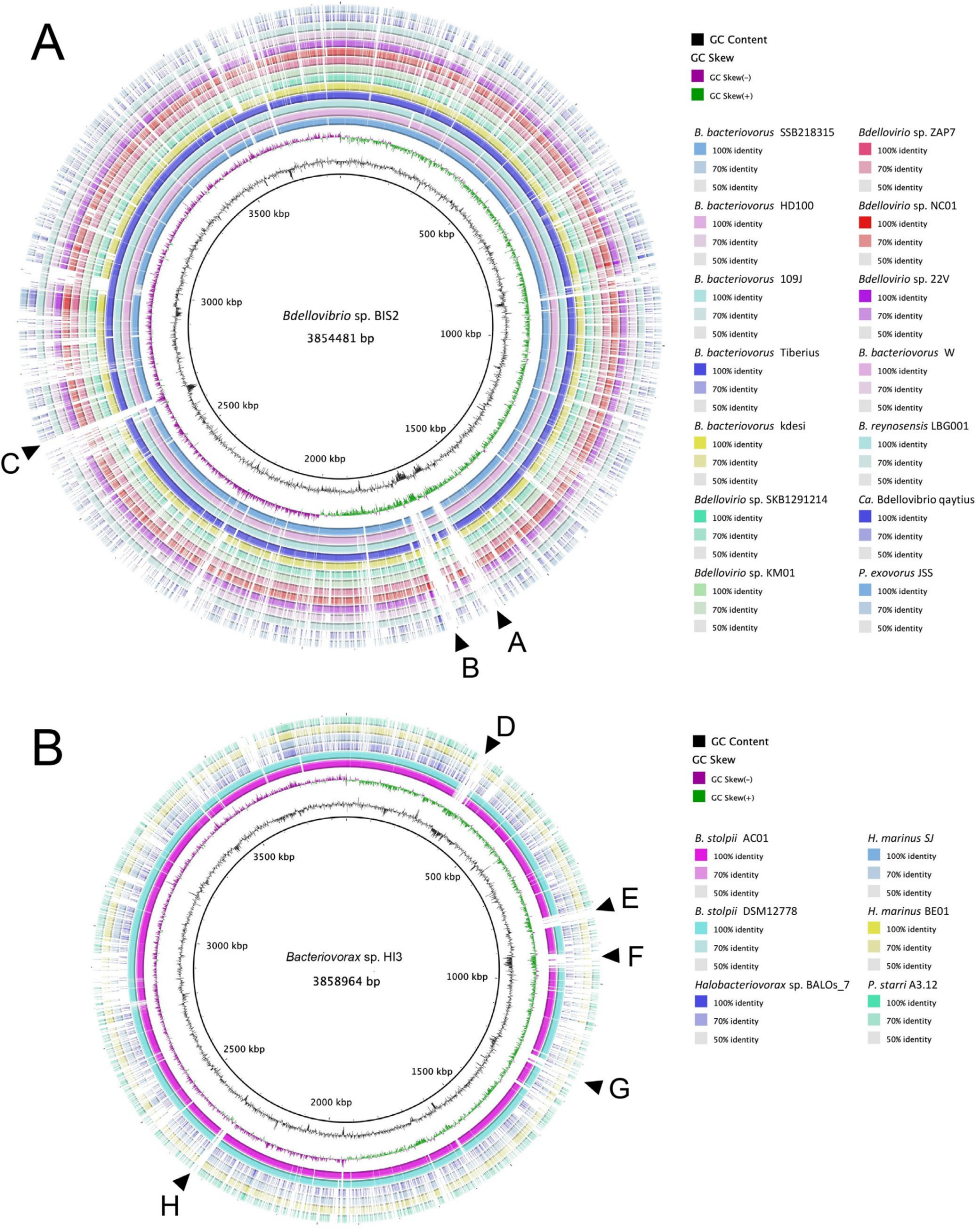
Genome‐wide comparisons of (A) *Bdellovibrionales* and (B) *Bacteriovoracales* nucleotide sequences. The genomes of (A) BIS2 and (B) HI3 are shown as the innermost ring. The next ring is a plot of the GC content and the purple and green rings show the GC skew, generated using BRIG with sliding windows to determine the deviation from the average value for the genome. Each individual alignment is shown as a block along the ring, and the colour gradation of the blocks reflects the percent similarity of alignments. Arrows highlight eight major regions (> 20 kbp) potentially unique to the reference strains: Three in BIS2 (regions A–C; coordinates: A, 1610–1640 kbp; B, 1674–1712 kbp; C, 2640–2665 kbp) and five in HI3 (regions D–H; coordinates: D, 333–373 kbp; E, 806–841 kbp; F, 920–973 kbp; G, 1207–1256 kbp; H, 2316–2340 kbp).

To represent phylogenetic diversity and genomic variation, the genomes of BIS2 and HI3 were compared with those of 14 *Bdellovibrionales* strains excluding *Micavibrio* and six *Bacteriovoracales* strains. Pairwise comparisons identified three genetic regions unique to BIS2 (regions A–C; Figures 4A, S7A) and five regions unique to HI3 (regions D–H; Figures 4B and S7B). Low coverage (12%–33%) but > 80% identity to related species within these regions implied specific evolutionary trajectories in the genomes of BIS2 and HI3. In particular, only < 20% of amino acid sequences in region E were aligned to *Bacteriovoracaceae* sequences, although all aligned to *Bdellovibrionota* sequences. Genes in this region might have been retained specifically in HI3 after divergence from the ancestor or acquired from other bacterial lineages (Hobley et al. 2012; Williams et al. 2019). In contrast, in BIS‐specific region C, only 72.2% of protein‐coding sequences were aligned to *Bdellovibrionota* sequences, and many genes had a high identity with those in non‐BALO bacteria in various habitats (Table S6), implying their acquisition from non‐BALO bacteria during divergence from the ancestor of *Bdellovibrionota*. Given that SSB218315 (most closely related to BIS2) is a soil isolate and the BIS2‐specific region contained four genes that were highly similar to those in a soil isolate, *Bdellovibrio* sp. BCCA, BIS2 might have diverged from soil BALOs and acquired genes to facilitate adaptation to the freshwater environment. Recent comparative genomic studies have also indicated that *Bdellovibrio* and *Halobacteriovorax* have evolved through horizontal gene transfer from non‐BALO bacteria (Pan et al. 2011; Enos et al. 2018), and the origin of the acquired genes often depends on the habitat (Hobley et al. 2012).

The BIS2‐ and HI3‐specific regions contained genes encoding hydrolases potentially related to their predatory lifestyle. For example, the BIS2‐specific region C contained an ATPase (BBIS2_24980), which may be involved in a membrane transport system or energy metabolism. Other specific regions also encoded enzymes likely contributing to prey cell component degradation, such as a lysozyme (region E: BHI3_07710) and metallopeptidase (region F: BHI3_08810). Such unique genes might have contributed to the distinct prey preferences among BALOs and eventually to their overall diversity in natural environments.

### 3.5 Genes Affecting Differential Predatory Properties in Strains BIS2 and HI3

**FIGURE 5:**
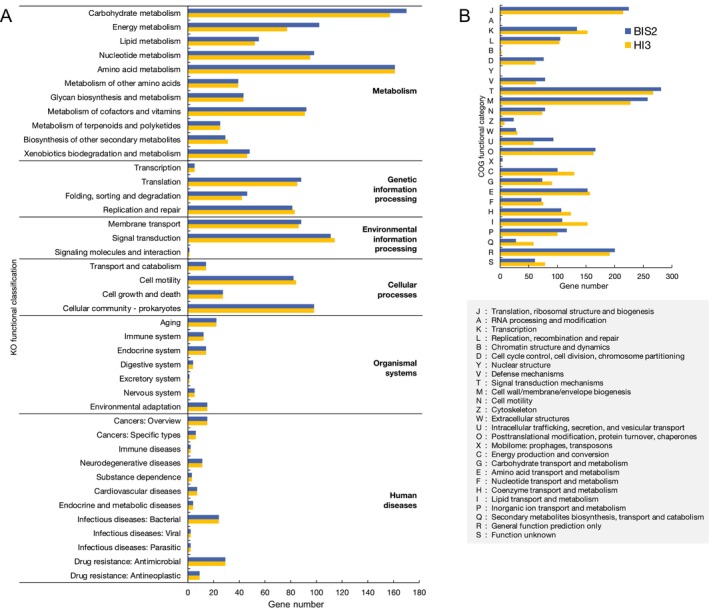
Functional categories of genes in the genomes of BIS2 and HI3. (A) KEGG and (B) COG functional annotation of BIS2 (blue) and HI3 (yellow) are shown. Genes assigned to multiple functional categories were counted in each respective category.

The functional classification of predicted genes in the BIS2 and HI3 genomes based on COG and KO categories is summarised in Figure 5. Their overall functional compositions were similar to each other, particularly in the KO classification. Whereas a previous study has shown that the functional composition in BALO genomes depends on phylogenetic affiliation (Davis et al. 2024), the observed similarity between BIS2 and HI3, belonging to different genera, suggested that the habitat is another crucial determinant of the genomic functional profiles of BALOs.

**FIGURE 6:**
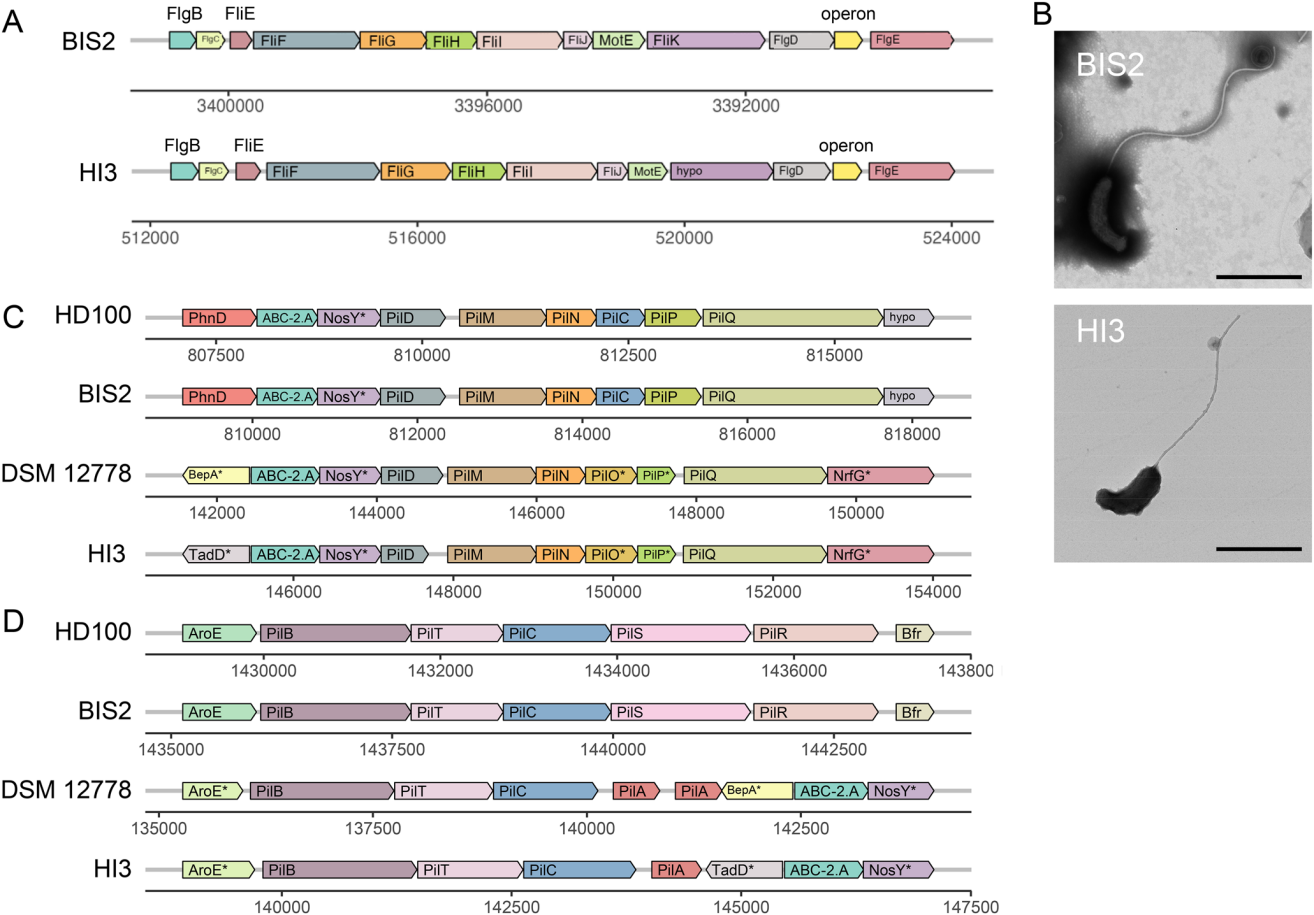
Comparison of flagellar and pilus assembly gene clusters in BIS2, HI3 and their close relatives. (A) Diagrammatic comparison of a gene cluster containing the predicted flagellar assembly genes in the KO and COG annotation in BIS2 and HI3. FlgB/C, flagellar basal‐body rod protein; FlgD, flagellar basal‐body rod modification protein; FlgE, flagellar hook protein; FliE, flagellar hook‐basal body complex; FliF, flagellar M‐ring protein; FliG, flagellar motor switch protein; FliH, flagellar assembly protein; FliI, flagellum‐specific ATP synthase; FliJ, flagellar operon protein; MotE, flagellar motility protein (hypothetical). (B) TEM images of BIS2 and HI3 cells. The scale bar indicates 1 μm. (C, D) Comparison of the pilus assembly gene cluster in BIS2, HI3, HD100, and DSM 12778. Loci (C) and (D) are contiguous in HI3. Asterisks (*) indicate that gene identification was based on COG annotation (not KO annotation). ABC‐2.A, ABC‐2 type transport system ATP‐binding protein; AroE, shikimate dehydrogenase; BepA, periplasmic chaperone/metalloprotease containing TPR domains; Bfr, bacterioferritin; NosY, ABC‐type transporter permease; NrfG, cytochrome c‐type biogenesis protein containing TPR repeats; PhnD, phosphate/phosphite/phosphonate ABC transporter substrate‐binding protein; PilA/B/C/D/M/N/O/P/Q, type IV pilus assembly protein; PilR, NtrC‐family two‐component system response regulator; PilS, NtrC‐family two‐component system sensor histidine kinase; PilT, twitching motility protein; TadD, Flp pilus assembly protein containing TPR repeats (hypothetical).

As physical attachment to prey cells is essential for BALO predation (Lambert et al. 2006), flagellum‐mediated motility impacts the predation efficiency substantially. The KO‐based annotation identified 38 and 33 flagellar assembly genes in BIS2 and HI3, respectively. Most of these genes were distributed across three distinct loci, of which two clusters had identical gene compositions in both strains. BIS2 harboured a complete set of flagellar assembly genes, including those encoding the flagellar hook, rod and ring, components of the filament's basal structure. HI3 lacked the *filK* gene ortholog, which regulates hook length during flagellar synthesis (Erhardt et al. 2010). Furthermore, TEM observations confirmed that the flagella were shorter in HI3 (mean: 2.10 μm) than in BIS2 (mean: 3.48 μm) (Figures 6B and S8). These findings collectively imply that the deficiency of a *filK* gene ortholog impacts the flagellum morphogenesis in HI3. Morehouse et al. (2011) reported that *B. bacteriovorus* possesses three contiguous stator pairs interacting with the flagellar rotor ring (FliG) to produce high‐speed flagellar rotation. Three such motor pairs were also found in the BIS2 genome, while only one corresponding motor pair was identified in the HI3 genome. Considering differences in the rate of expansion of the lytic zone among the two BALOs in predation assays on prey colony (Figure 2B) and the directly measured swimming speeds (Figure S4 and Video S1), the number of flagellar motor units likely has a substantial impact on BALO motility.

TFP localised in the cell envelope is required for initial attachment to and invasion of prey cells (Chanyi and Koval 2014; Avidan et al. 2017). The composition and genomic localization of regions containing genes encoding the TFP assembly were compared among BIS2, HI3, and type strains of their respective genera, HD100 and *Bacteriovorax stolpii* DSM 12778. The TFP gene clusters were highly conserved within each genus (i.e., identical in BIS2 and HD100 and nearly identical in HI3 and DSM 12778). Notable differences were found among different genera. TFP gene clusters in HI3 and DSM 12778 were co‐localised in a single large genomic region (ca. 13 kbp), while the homologous genes in BIS2 and HD100 were located in two distant regions (Figure 6C,D). These results suggest that significant genomic rearrangements occurred during the divergence of the *Bacteriovorax* and *Bdellovibrio* lineages from a common ancestor. Within the TFP assembly, PilC functions as an ATPase that powers the extension and retraction of TFP and is an essential attachment factor for *Neisseria* to infect human cells (Kirchner and Meyer 2005). In addition, amino‐acid sequence variants, particularly at the N‐terminus, can influence attachment properties (Morand et al. 2001). The PilC sequence was highly similar in BIS2 and HD100 (98% identity, 100% coverage), with no significant similarity between BIS2 and HI3. The variation in the TFP assembly may contribute to the distinct prey spectra of BIS2 and HI3 (Figure 1).

BALOs must secrete hydrolytic enzymes to degrade various cell components and thereby to invade and prey on target cells. The cell envelope of Gram‐negative bacteria consists mainly of phospholipids and lipopolysaccharide, which exhibit considerable diversity in molecular structure and function across species (Raetz and Whitfield 2002; Leus et al. 2023). Therefore, BALOs require a suite of hydrolases to sequentially breach outer membrane structures and access the periplasm in prey cells. We observed differences in the type and number of predicted hydrolase‐encoding genes between BIS2 and HI3 (Table S7). BIS2 uniquely encodes an α‐amylase (BBIS2_11100) that hydrolyses α‐glycosidic bonds in polysaccharides, whereas HI3 exclusively encodes a phospholipase A_2_ (BHI3_24000) that hydrolyses ester bonds in glycerophospholipids (Lin et al. 2018). Such differences in hydrolytic enzymes likely lead to distinct prey ranges and predation efficiencies. Beyond these general differences, BIS2 and HI3 also had differences in specific hydrolases, which are key determinants of predatory phenotypes (Caulton and Lovering 2023). BIS2 possessed two genes predicted to encode *N‐*acetylglucosamine (GlcNAc) deacetylase, similar to HD100. HI3 contained five genes encoding this enzyme. GlcNAc deacetylase hydrolyses N‐C bonds and contributes to the decomposition of the prey cell envelope. Notably, its interruption in *B. bacteriovorus* prevents the destruction of the prey cell wall, resulting in empty prey membranes or prey ghost (Lambert et al. 2016). Furthermore, BIS2 possessed a gene (BBIS2_02860) encoding a protein with high similarity (94.88% identity, 100% coverage) to DslA (Bd0314 in HD100). However, no ortholog of DslA was identified in HI3. DslA is a lysozyme that acts upon GlcNAc deacetylated peptidoglycan and as a signal controlling the timing of BALO exit from prey cells (Harding et al. 2020). This variation in hydrolytic enzymes is likely related to the differences in predation phenotypes noted between BIS2 and HI3 (i.e., the incomplete prey lysis and “prey ghost” formation by BIS2, in contrast to the complete lysis by HI3, as well as the differences in their lytic zone expansion rates) (Figures 2, 3, and S3). These findings suggest that the BALO strains exhibit distinct predation phenotypes with a genetic basis.

BIS2 and HI3 also differed in the central metabolic pathways. BIS2 possessed complete pathways for both glycolysis and the TCA cycle, while HI3 lacked the gene for pyruvate oxidation, a key process connecting glycolysis to the TCA cycle (Figure S9). Furthermore, HI3 possessed a fatty acid elongation pathway, which was absent in BIS2. HI3 also harboured a significantly higher number of genes assigned to COG category I (lipid transport and metabolism) than that in BIS2. These genomic differences suggest that HI3 possesses a distinct genetic potential for lipid metabolism, which could affect the extent to which these BALOs degrade different parts of the prey cell. Differences in COG category I could affect the extent to which BALOs degrade different parts of the prey cell. Moreover, variation in metabolic pathways may contribute to determining the growth rate, yield, or overall energetic costs associated with the predatory lifecycle of BIS2 and HI3 (Noor et al. 2016). Despite providing a comprehensive comparison of the genomic features of BIS2 and HI3, this study still has limitations. The findings obtained in this study rely on genomic predictions and a lack of direct functional validation of gene expression or protein activity. Furthermore, numerous hypothetical proteins with unestablished functions in BALOs (22%–55% of total CDS; Table S3) make it challenging to clarify their genetic predatory characteristics. Future studies employing experimental genetics and broader comparative analyses across more diverse BALO strains will increase our understanding of the molecular mechanisms governing their predatory phenotypes.

## 4 Conclusions

Our integrated analysis of predatory phenotypes and genomic features suggests that coexisting BALOs partition their niche through contrasting predatory traits and distinct genetic machinery. One BALO strain evaluated in this study, BIS2, displayed rapid spatial exploration at the cost of incomplete prey lysis, while the other strain, HI3, adopted a more localised predation strategy but completely lysed prey, enabling greater growth. These distinct predation properties were corroborated by significant differences at the genetic level, particularly in the gene sets affecting motility, prey recognition, and lytic capabilities. Furthermore, the discovery of genomic regions unique to each predator highlighted the divergent evolutionary trajectories leading to diverse predatory properties among BALOs. The coexistence of BALOs with distinct predation strategies can exert multifaceted predatory pressures in natural microbial ecosystems, thereby shaping the bacterial community structure and dynamics. Unravelling these diverse predation strategies is essential for understanding the complex interaction network that governs the structure and stability of microbial communities. Further studies validating the competitive dynamics of BALOs in shared environments, along with research linking predatory phenotypes to genotypes of environmental BALOs will deepen our understanding of the evolution and ecology of BALOs and unlock the potential for broader biotechnological applications through leveraging the variety of predatory properties.

## Author Contributions

**Tomomi Sugiyama:** conceptualization, data curation, formal analysis, investigation, methodology, validation, visualisation, writing – original draft. **Tsubasa Kojima:** investigation. **Naoki A. Uemura:** investigation, methodology, writing – review and editing. **Daisuke Nakane:** investigation, methodology, writing – review and editing. **Hideo Dohra:** formal analysis, writing – review and editing. **Daisuke Inoue:** conceptualization, funding acquisition, project administration, resources, validation, supervision, writing – review and editing. **Michihiko Ike:** funding acquisition, project administration, resources, writing – review and editing.

## Funding

This work was supported by Institute for Fermentation, Osaka (G‐2021‐3‐049), Kurita Water and Environment Foundation (22T009), Science and Technology Research Partnership for Sustainable Development (JPMJSA2004), Japan Science and Technology Agency (JPMJSP2138), and Japan Society for the Promotion of Science (JP24K21902).

## Conflicts of Interest

The authors declare no conflicts of interest.

## Supporting information


**Data S1:** emi70243‐sup‐0001‐FigureS1–S9.docx.

**Data S2:** emi70243‐sup‐0002‐TextS1–S2.docx.

**Data S3:** emi70243‐sup‐0003‐VideoS1.mp4.

**Video S1:** Representative video showing the swimming behaviors of HI3 (left panel) and BIS2 (right panel). Cells were visualized using dark‐field microscopy. The time is displayed in seconds. The field of view corresponds to 188 × 141 µm.

## Data Availability

All genetic sequences are available at the DDBJ/ENA/GenBank database. The complete genome sequence of *Bdellovibrio* sp. BIS2 determined in this study has been deposited at DDBJ/ENA/GenBank under accession no. AP035879, with link to BioProject accession no. PRJDB18512 and BioSample accession no. SAMD00802424. All other data will be made available on request.
