# Supplementary material for: Comparison of Predatory Phenotypes and Genotypes Between Bdellovibrio sp. BIS2 and Bacteriovorax sp. HI3 Isolated From the Same Freshwater Environment
Source: Environ Microbiol. 2026 Jan 22;28(1):e70243. doi: 10.1111/1462-2920.70243 (PMC12827227; doi:10.1111/1462-2920.70243)
Supplement: Supplementary file 1 — Data S1: emi70243‐sup‐0001‐FigureS1–S9.docx. [file EMI-28-e70243-s002.docx]

**Supplementary Figures**


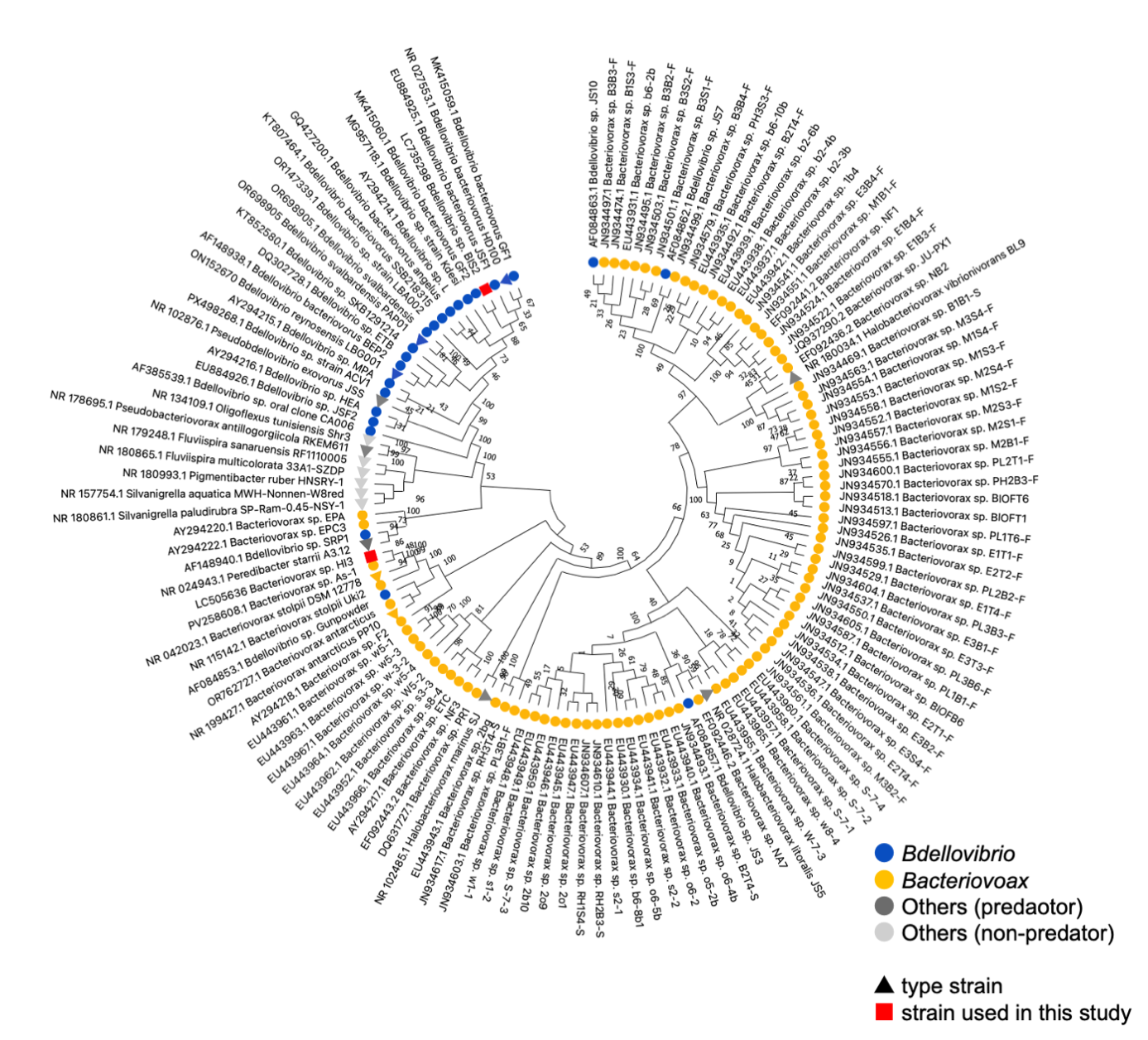


**Fig. S1** Phylogenetic tree based on 16S rRNA sequences for predatory strains belonging to *Bdellovibrio* and *Bacteriovorax* and other type strains belonging to *Bdellovibrionota*. Sequences were retrieved from the GenBank database in December 2025. To ensure dataset quality and reduce redundancy, sequences shorter than 1,400 bp were excluded, and unique representative sequences were selected using a 99.5% identity threshold (i.e., only one strain was selected for groups sharing >99.5% identity). Evolutionary relationships were inferred using RaxML. The numbers at the nodes indicate the gene support index.

**
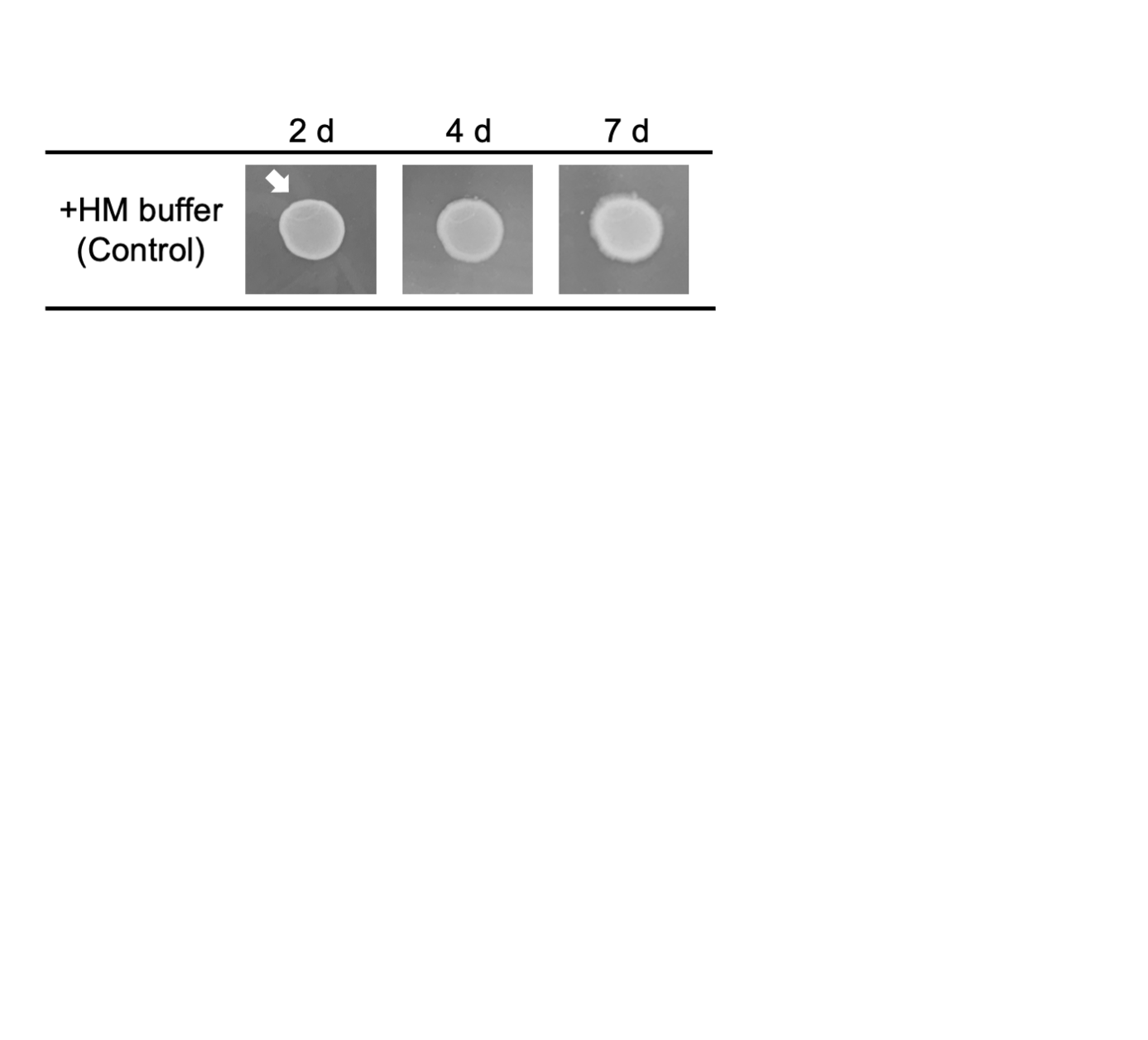
**

**Fig. S2** Negative control (buffer only) for the predation assay on nutrient agar. The white arrow indicates the inoculation point of the HM buffer. No lytic zone was observed throughout the incubation period.


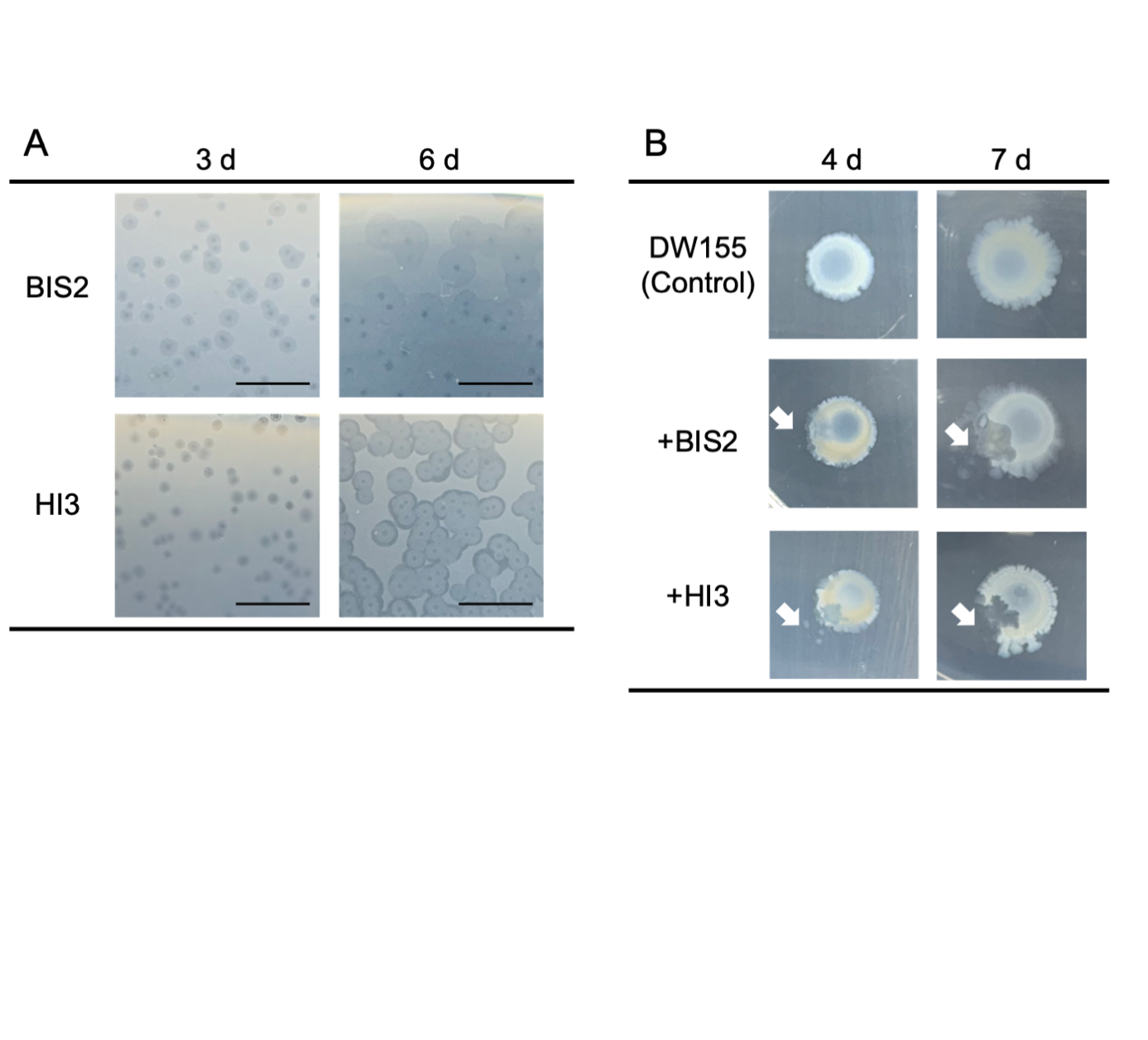


**Fig. S3** Predation assays using DW155 as prey. (A) Plaque growing on a double-layer agar plate. The scale bar indicates 1 cm. (B) Predation on a bacterial colony on the surface of the nutrient agar. White arrow shows the BALO inoculation point.


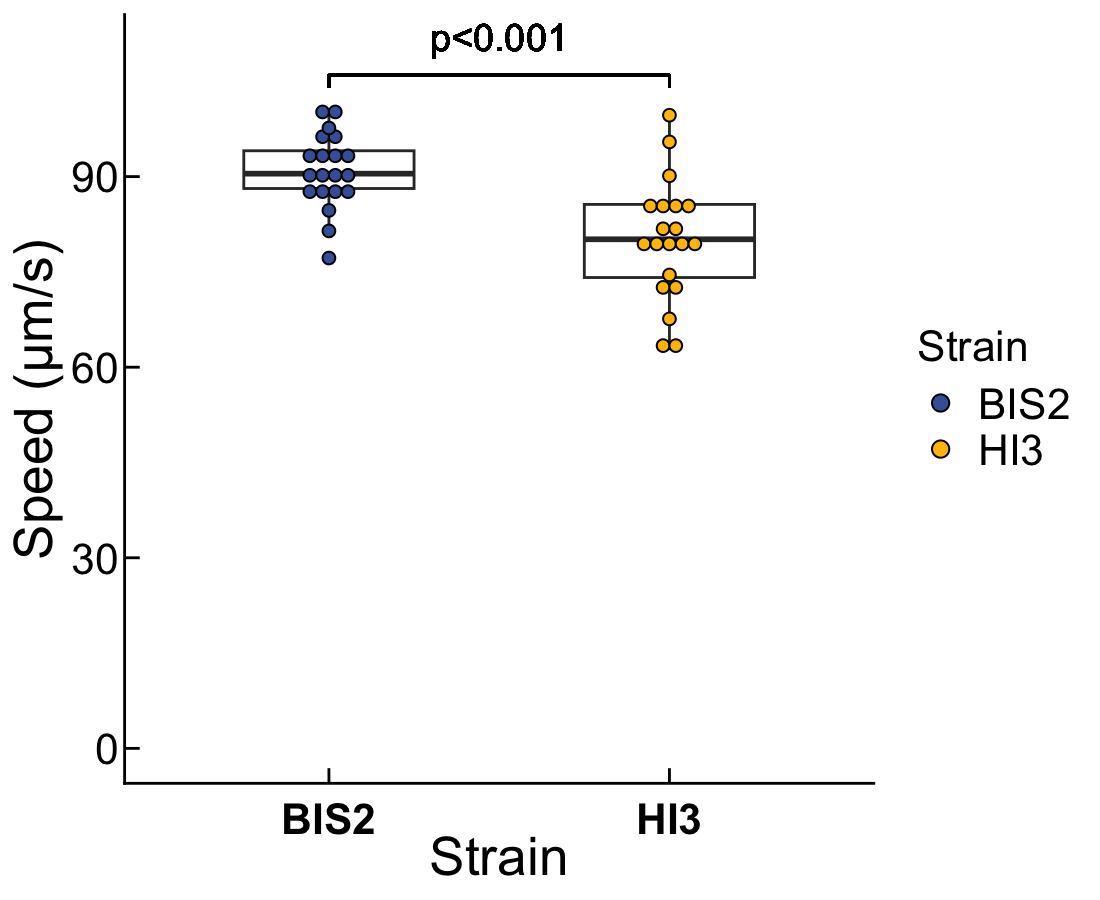


**Fig. S4** Box plot comparing the swimming speed between BIS2 and HI3. Individual data points are overlaid on the box plots (n=20). The boxes represent the interquartile range between the first and third quartiles (25th and 75th percentiles, respectively) and the horizontal line inside the box defines the median. The p value in the figure was obtained from the Welch's t-test.

**Fig. S5** Phylogenetic tree based on housekeeping genes (81 genes) and ANI heat maps based on whole genomes for predatory strains belonging to *Bdellovibrionota*. The tree was generated using the UBCG2 pipeline with the concatenated sequences of 81 universal bacterial core genes. Evolutionary relationships were inferred using RaxML. The numbers at the nodes indicate the gene support index. The scale bar represents 0.50 nucleotide substitutions per site. The ANI values were calculated with the ANI/AAI-Matrix (Kostas lab, Atlanta, GA, USA) using BLAST.


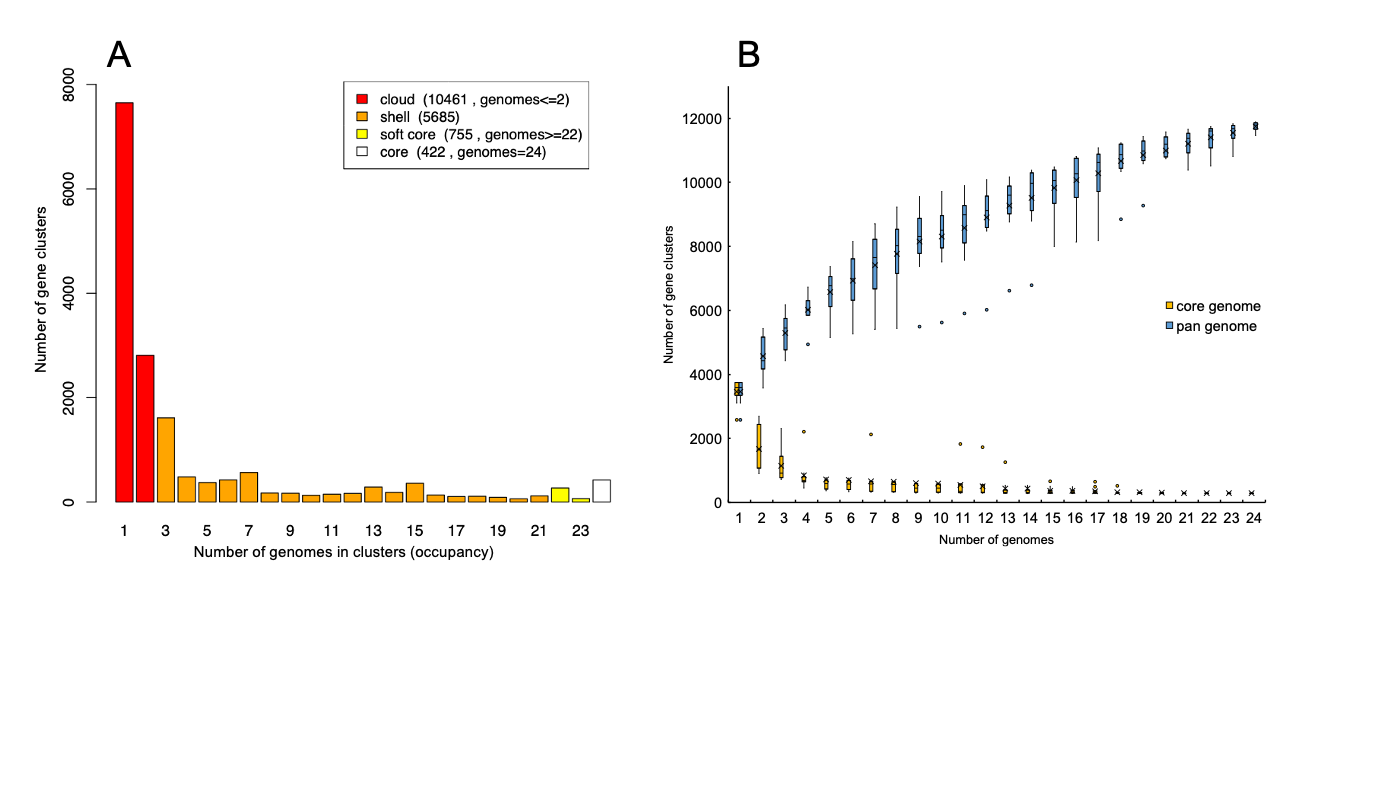


**Fig. S6** Core and pan-genomes of predatory strains belonging to *Bdellovibrionota*. (A) Bar plot showing the absolute size frequencies of orthologous clusters as predicted using the OMCL algorithm. The pangenome was made up of 16,384 genes. (B) Total number of genes or pan-genome (blue) and shared or core-genome (yellow) for 23 *Bdellovibrionota* strains. The orthologues clusters were predicted using the BDBH algorithm. The pangenome comprised 11,882 genes while the core genomes comprised 287 genes.

**Fig. S7** Genome synteny plots comparing (A) BIS2 with its closest relative, *Bdellovibrio bacteriovorus* SSB218315, and (B) HI3 with its closest relative, *Bacteriovorax stolpii* AC01. Alignments were generated using bl2seq and visualized using GenomeMatcher.


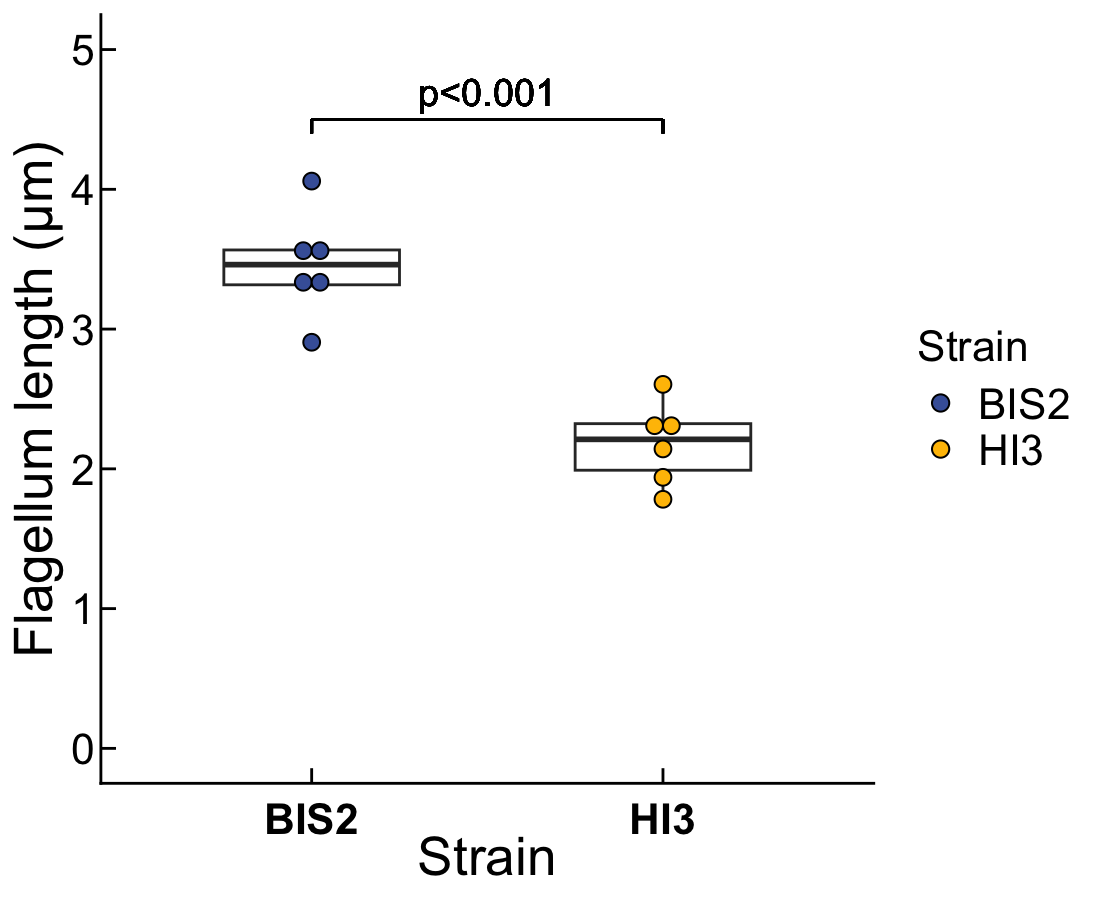


**Fig. S8** Box plot showing flagellar lengths of BIS2 and HI3. Individual data points are overlaid on the box plots (n = 6). The boxes represent the interquartile range between the first and third quartiles (25th and 75th percentiles, respectively) and the horizontal line inside the box defines the median. The p value in the figure was obtained from the Student's t-test.

**Fig. S9** KEGG pathway maps related to glycolysis/gluconeogenesis for (A) BIS2 and (B) HI3. Genes associated to this pathway in the KEGG database are highlighted in green. Complete pathway modules identified using KEGG Mapper are shown in red.
