# Supplementary material for: Comparison of Predatory Phenotypes and Genotypes Between Bdellovibrio sp. BIS2 and Bacteriovorax sp. HI3 Isolated From the Same Freshwater Environment
Source: Environ Microbiol. 2026 Jan 22;28(1):e70243. doi: 10.1111/1462-2920.70243 (PMC12827227; doi:10.1111/1462-2920.70243)
Supplement: Supplementary file 2 — Data S2: emi70243‐sup‐0002‐TextS1–S2.docx. [file EMI-28-e70243-s004.docx]

**Supplementary Texts**

**Text S1. Isolation of *Bdellovibrio* sp. BIS2**

*Bdellovibrio* sp. BIS2 was isolated from surface water of a freshwater pond at The University of Osaka (34°82′ N, 135°53′E), where *Bacteriovorax* sp. HI3 was also isolated. The water sample was collected on August 22, 2022 and passed twice through a 3-µm membrane filter (Merck Millipore, Darmstadt, Germany) to remove coarse particles. The filtrate was concentrated by centrifugation (20,000 *×g*, 4 °C, 10 min), washed twice with HM buffer, and filtered twice through a 0.45-µm filter (Advantec, Tokyo, Japan), after which 20 mL of the filtrate was transferred to a 50-mL glass vial. *Methylophillus* sp. DW102 (Ishizawa *et al.*, 2020). The prey used was precultivated, harvested by centrifugation (20,000 *×g*, 4 °C, 10 min) and washed twice with HM buffer. The resulting prey cells were inoculated to the aforementioned filtrate in a glass vial to obtain a final optical density at an OD_600_ of 1. Then, the co-culture was incubated at 28 °C with rotary shaking (120 rpm) for 6 days to enrich potential BALOs in the collected samples. A control culture, where the filtrate in the glass vial was sterilized through autoclaving prior to the inoculation of prey cells, was also prepared to confirm that the OD_600_ decline in the co-culture could be attributed to the biolytic activity in the prepared filtrate.

The co-culture showing a significant OD_600_ decline compared with values for the control was selected for the isolation of BALOs using a double-layer agar plating technique (Jurkevitch, 2012). The culture was filtered twice through a 0.45-µm filter and serially diluted tenfold with sterile HM buffer. Then, 100 µL of the dilution was mixed with 300 µL of the DW102 suspension (OD_600_ = 30) and overlaid onto a double-layer agar plate. The plates were incubated statically at 28 °C for 5 days, and the formation of lytic halos was monitored. Once lytic halos were found, an agar plate segment with a single lytic halo was collected and homogenized using BioMasher II (Nippi, Tokyo, Japan). The homogenate was repeatedly subcultured onto fresh double-layer agar plates prepared with *Escherichia coli* HB101 as the prey. After the purity was confirmed, the isolated BALO strain, designated BIS2, was stored in 25% glycerol at -80 °C.

**Text S2. Identification and genome annotation of *Bdellovibrio* sp. BIS2**

Whole genome sequencing was performed for BIS2. For the preparation of biomass to obtain genomic DNA, a segment of a double-layer agar plate with a lytic halo that formed following the predation of HB101 by BIS2 was picked up and homogenized. The homogenate was inoculated in R2A medium containing HB101 as the prey and cultivated at 28 °C with rotary shaking (120 rpm) for 3 days. The resulting culture was filtered twice through a 0.45-µm filter to remove the residual prey cells. The genomic DNA was extracted from BIS2 cells in the filtrate using the NucleoBond HMW DNA Kit (Macherey-Nagel, Düren, Germany). A library was prepared using SMRTbell Prep Kit 3.0 (Pacific Biosciences, Menlo Park, CA, USA) and sequenced using PacBio HiFi sequencing and the PacBio Sequel II system (Pacific Biosciences). The library construction and sequencing were performed at Macrogen, Inc. (Seoul, Republic of Korea). PacBio HiFi reads with a length of ≥7.5 kb were extracted using SeqKit v. 0.8.0 (Shen *et al.*, 2016). The selected reads were used for *de novo* genome assembly using Flye v. 2.9.1 (Kolmogorov *et al.*, 2019) (parameter: genome size, 3.8 Mb). The resulting single contig was manually rotated using Geneious Prime v. 2023.0 (Kearse *et al.*, 2012) to place the *dnaA* gene at the beginning of the circular chromosome sequence. Gene prediction and functional annotation were performed using DFAST-core v. 1.2.16 (Tanizawa *et al.*, 2018), using MetaGeneAnnotator v. 2008/08/19 (Noguchi *et al.*, 2008) or GeneMarkS2 v. 1.14_1.25 (Lomsadze *et al.*, 2018) for predicting protein-coding sequences (CDSs), RNAmmer v. 1.2 (Lagesen *et al.*, 2007) for rRNA genes, and tRNAscan-SE v. 2.0.5 (Chan *et al.*, 2021) for tRNA genes.

The complete genome of BIS2 consisted of a single circular chromosome with a size of 3,854,481 bp and a G+C content of 50.4%. These estimates were generally similar to those of *Bdellovibrio bacteriovorus* 109J and relatively high among BALOs (averages: ~3.8 Mbp, ~45%). The BIS2 genome contained 36 predicted tRNA genes and two copies each of 5S, 16S, and 23S rRNA genes but lacked CRISPR loci. A total of 3,645 CDSs were predicted, of which 807 were annotated as hypothetical proteins. The complete genome sequence of BIS2 has been deposited at DDBJ/ENA/GenBank under accession no. AP035879.

Phylogenetic and average nucleotide identity (ANI) analyses were conducted based on the complete genomes of 24 BALO strains (Table S3), as described in the main text (Section 2.7). The genome of BIS2 exhibited the highest similarity (97.4% ANI value) to that of *Bdellovibrio bacteriovorus* SSB218315, a periplasmic BALO isolated from soil under banana plants in Mexico using *Salmonella* sp. as prey (Oyedara *et al.*, 2016). The ANI value between BIS2 and the type strain *B. bacteriovorus* HD100 was 89.3%, which is significantly below the 95% threshold frequently used standard for species delineation (Konstantinidis and Tiedje, 2007). Therefore, BIS2 was classified within the family *Bdellovibrionaceae* and designated *Bdellovibrio* sp. BIS2.

**References**

Balebona, M.C., M.J. Andreu, M.A. Bordas, I. Zorrilla, M.A. Moriñigo, and J.J. Borrego. 1998. “Pathogenicity of Vibrio alginolyticus for cultured gilt-head sea bream (Sparus aurata L.)” *Applied and Environmental Microbiology* **64**: 4269–4275.

Caccavo, F., D.J. Lonergan, D.R. Lovley, M. Davis, J.F. Stolz, and M.J. McInerney. 1994. “*Geobacter sulfurreducens* sp. nov., a hydrogen- and acetate-oxidizing dissimilatory metal-reducing microorganism” *Applied and Environmental Microbiology* **60**: 3752–3759.

Chan, P.P., B.Y. Lin, A.J. Mak, and T.M. Lowe. 2021. “TRNAscan-SE 2.0: improved detection and functional classification of transfer RNA genes” *Nucleic Acids Research* **49**: 9077–9096.

Inoue, D., N. Hiroshima, S. Nakamura, H. Ishizawa, and M. Ike. 2022. “Characterization of two novel predatory bacteria, *Bacteriovorax stolpii* HI3 and *Myxococcus* sp. MH1, isolated from a freshwater pond: prey range, and predatory dynamics and efficiency” *Microorganisms* **10**: 1816.

Inoue, D., H. Tsutsui, Y. Yamazaki, K. Sei, S. Soda, M. Fujita, and M. Ike. 2008. “Application of real-time polymerase chain reaction (PCR) coupled with ethidium monoazide treatment for selective quantification of viable bacteria in aquatic environment” *Water Science and Technology* **58**: 1107–1112.

Ishizawa, H., M. Tada, M. Kuroda, D. Inoue, H. Futamata, and M. Ike. 2020. “Synthetic bacterial community of duckweed: a simple and stable system to study plant-microbe interactions” *Microbes and Environments* **35**: ME20112.

Jenni, B., L. Realini, M. Aragno, and A.Ü. Tamer. 1988. “‘Taxonomy of non H_2_-lithotrophic, oxalate-oxidizing bacteria related to *Alcaligenes eutrophus*. *Systematic and Applied Microbiology* **10**: 126–133.

Jurkevitch, E. 2012. ”Isolation and classification of *Bdellovibrio* and like organisms.” *Current Protocols in Microbiology* **26**: Unit 7B.1.

Kearse, M., R. Moir, A. Wilson, S. Stones-Havas, M. Cheung, S. Sturrock, et al. 2012. “Geneious Basic: an integrated and extendable desktop software platform for the organization and analysis of sequence data” *Bioinformatics* **28**: 1647–1649.

Kolmogorov, M., J. Yuan, Y. Lin, and P.A. Pevzner. 2019. “Assembly of long, error-prone reads using repeat graphs” *Nature Biotechnology* **37**: 540–546.

Konstantinidis, K.T. and J.M. Tiedje. 2007. “Prokaryotic taxonomy and phylogeny in the genomic era: advancements and challenges ahead” *Current Opinion in Microbiology* **10**: 504–509.

Lagesen, K., P. Hallin, E.A. Rødland, H.-H. Staerfeldt, T. Rognes, and D.W. Ussery. 2007. “RNAmmer: consistent and rapid annotation of ribosomal RNA genes” *Nucleic Acids Research* **35**: 3100–3108.

Lomsadze, A., K. Gemayel, S. Tang, and M. Borodovsky. 2018. “Modeling leaderless transcription and atypical genes results in more accurate gene prediction in prokaryotes” *Genome Research* **28**: 1079–1089.

Noguchi, H., T. Taniguchi, and T. Itoh. 2008. “MetaGeneAnnotator: detecting species-specific patterns of ribosomal binding site for precise gene prediction in anonymous prokaryotic and phage genomes” *DNA Research* **15**: 387–396.

Oyedara, O.O., E.J. De Luna-Santillana, O. Olguin-Rodriguez, X. Guo, M.A. Mendoza-Villa, J.L. Menchaca-Arredondo, et al. 2016. “Isolation of *Bdellovibrio* sp. from soil samples in Mexico and their potential applications in control of pathogens” *MicrobiologyOpen* **5**: 992–1002.

Salanoubat, M., S. Genin, F. Artiguenave, J. Gouzy, S. Mangenot, M. Arlat, et al. 2002. “Genome sequence of the plant pathogen *Ralstonia solanacearum*” *Nature* **415**: 497–502.

Semwal, A., A. Kumar, and N. Kumar. 2023. “A review on pathogenicity of *Aeromonas hydrophila* and their mitigation through medicinal herbs in aquaculture” *Heliyon* **9**: e14088.

Shen, W., S. Le, Y. Li, and F. Hu. 2016. “SeqKit: a cross-platform and ultrafast toolkit for FASTA/Q file manipulation” *PLOS One* **11**: e0163962.

Tanizawa, Y., T. Fujisawa, and Y. Nakamura. 2018. “DFAST: a flexible prokaryotic genome annotation pipeline for faster genome publication” *Bioinformatics* **34**: 1037–1039.

Tóth, Á., R. Máté, J. Kutasi, I. Bata-Vidács, E. Tóth, A. Táncsics, et al. 2021. “*Cellvibrio polysaccharolyticus* sp. nov., a cellulolytic bacterium isolated from agricultural soil” *International Journal of Systematic and Evolutionary Microbiology* **71**: 10.1099/ijsem.0.004805.

Xu, F., X. Zeng, Y. Gong, and Z. Shao. 2024. “Thiosulfate oxidation and autotrophy potential by marine prevalent heterotrophic bacteria of genus *Marinobacter*” *Acta Oceanologica Sinica* **43**: 89–97.

Zhang, X., G. Feng, Y. Liu, J. Li, X. Deng, Q. Yao, and H. Zhu. 2023. “Characterization of phytopathogen-preying Hyalangium versicolor sp. nov., and proposal for the reclassification of Cystobacter gracilis as Hyalangium gracile comb. Nov” *Archives of Microbiology* **205**: 198.
