# Supplementary material for: Comparison of Predatory Phenotypes and Genotypes Between Bdellovibrio sp. BIS2 and Bacteriovorax sp. HI3 Isolated From the Same Freshwater Environment
Source: Environ Microbiol. 2026 Jan 22;28(1):e70243. doi: 10.1111/1462-2920.70243 (PMC12827227; doi:10.1111/1462-2920.70243)
Supplement: Supplementary file 4 — Video S1: Representative video showing the swimming behaviors of HI3 (left panel) and BIS2 (right panel). Cells were visualized using dark‐field microscopy. The time is displayed in seconds. The field of view corresponds to 188 × 141 µm. [file EMI-28-e70243-s001.docx]

**Supplementary video caption**

Video S1. Representative video showing the swimming behaviors of HI3 (left panel) and BIS2 (right panel). Cells were visualized using dark-field microscopy. The time is displayed in seconds. The field of view corresponds to 188 × 141 µm.
